# Cortical activation during the verbal fluency task for obstructive sleep apnea patients with depressive symptoms: A multi‐channel fNIRS study

**DOI:** 10.1002/brb3.70038

**Published:** 2024-09-29

**Authors:** Xuan Zhang, Ning Zhang, Yang Yang, Shuo Wang, Ping Yu, Chun‐Xue Wang

**Affiliations:** ^1^ Department of Neurology, Beijing Tian Tan Hospital Capital Medical University Beijing China; ^2^ Department of Neuropsychiatry & Behavioral Neurology and Clinical Psychology, Beijing Tian Tan Hospital Capital Medical University Beijing China

**Keywords:** depressive symptoms, functional near‐infrared spectroscopy, obstructive sleep apnea, polysomnography

## Abstract

**Study objective:**

The aim of our study was to elucidate differences in brain activity patterns among obstructive sleep apnea (OSA) patients, OSA patients with depressive symptoms, and healthy controls (HCs). We also investigated the relationship between brain function and depression in OSA patients.

**Methods:**

A total of 95 subjects were included in the study, including 34 OSA patients without depressive symptoms, 31 OSA patients with depressive symptoms, and 30 HCs. The 53‐channel functional near‐infrared spectroscopy (fNIRS) was used to monitor the concentration of oxy‐hemoglobin (Oxy‐Hb) in the brain, whereas the participants performed the verbal fluency task, and the degree of depression was scored using the 17‐item Hamilton Rating Scale for Depression (HAMD‐17). Hierarchical regression models were conducted to analyze the association of fNIRS features with depressive symptom.

**Results:**

The Oxy‐Hb changes of the three groups were significantly different in Channels 25 (*H* = 9.878, *p* = .007) and 43 (*H* = 6.957, *p* = .031). Inter‐group comparisons showed that the Oxy‐Hb change of Channel 25 (located in the dorsolateral prefrontal cortex [DLPFC]) in OSA group was less than that in HC group (*p* = .006), and the Oxy‐Hb change of Channel 43 (located in the right frontal polar region) in OSA group with depression was less than that in OSA group (*p* = .025). Spearman's test showed that there was a significant negative correlation between HAMD‐17 scores and mean Oxy‐Hb changes in Channel 43 (*r* = −.319, *p* < .05) in the OSA patients. Using hierarchical regression, Oxy‐Hb changes in Channel 43 accounted for a significant proportion of the variation in outcome variables, even when accounting for other polysomnography features.

**Conclusions:**

Changes in the hemodynamic response of DLPFC may be a potential mechanism of executive dysfunction in OSA patients. And the right frontal polar region may be significant in assessing depressive symptoms in patients with OSA.

## Graphical abstract

Our study investigated the relationship between brain function and depression in OSA patients. The functional near‐infrared spectroscopy was used to monitor the concentration of Oxy‐Hb in the brain, whereas the participants performed the verbal fluency task, and the degree of depression was scored using the 17‐item Hamilton Rating Scale for Depression. The right frontal polar region may be significant in assessing depressive symptoms in patients with OSA.

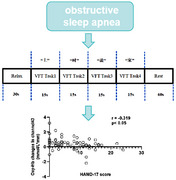

## 1 BRIEF SUMMARY

### 1.1 Current knowledge/study rationale

Depressive symptoms are a common clinical manifestation in obstructive sleep apnea (OSA) patients, but how brain function changes affect the occurrence of depressive symptoms in OSA patients remains unclear. The aim of our study was to elucidate differences in brain activity patterns among OSA patients without depressive symptoms, OSA patients with depressive symptoms, and healthy controls, and to explore the relationship between brain function and depression in OSA patients.

### 1.2 Study impact

Our study is the first to use functional near‐infrared spectroscopy (fNIRS) to demonstrate differences in prefrontal activation during verbal fluency task (VFT) between healthy subjects and OSA patients, showing that altered prefrontal hemodynamic responses may be a potential mechanism for executive dysfunction in OSA patients. At the same time, this study provides some evidence for fNIRS as a potential tool for predicting depressive symptoms in OSA patients. Our study provides neurological evidence for executive function in OSA and suggests an association between altered brain function and depressive symptoms.

## 2 INTRODUCTION

OSA is a common disorder characterized by recurrent episodes of partial or complete collapse of the upper airway during sleep, resulting in reduced or absent airflow lasting for at least 10 s and associated with long‐term health consequences (Gottlieb & Punjabi, 2020; Landry et al., 2023). Depression is common in people with OSA, and a recent meta‐analysis showed that the prevalence of depression in OSA patients was 35% (Garbarino et al., 2020). Although the problem of OSA with depression has received increasing attention, the relationship between OSA and depression is still poorly understood, and few studies have explored its neurophysiological mechanism (Morrone et al., 2022). And at the time of the first depressive episode, most patients will have significant cognitive impairment, leading to deterioration of life quality, but the mechanism of cognitive impairment in depression is unclear (Cambridge et al., 2018; Cullen et al., 2023). There is growing evidence that OSA is usually associated with mild impairment of attention, memory, and executive function (Bubu et al., 2020; Marchi et al., 2023; Sabil & Gagnadoux, 2023). Abnormal neural activity in some brain regions of OSA children may be the neural mechanism of impaired cognitive function (Ji et al., 2021). However, its various comorbidities, such as Alzheimer's disease and Parkinson's disease, are also known to cause cognitive impairment, and its own role in cognitive decline remains controversial (Gosselin et al., 2019; Osorio et al., 2022).

The combination of neuropsychological tasks with brain imaging techniques can provide a real‐time view of the patient's brain function during the execution. fNIRS is a safe and noninvasive neuroimaging technique applicable to a wide range of populations. It uses near‐infrared light (650–1000 nm) to measure the concentrations of oxy‐hemoglobin (Oxy‐Hb) and deoxy‐hemoglobin (Deoxy‐Hb), which can estimate the functional brain activity under different cognitive tasks (Yeung & Lin, 2021). And it has better spatial resolution than electroencephalography (EEG) and better temporal resolution than functional magnetic resonance imaging (fMRI). The fNIRS system measures hemodynamic signals from the surface of the cortex with a spatial resolution of 2–3 cm, allowing for more accurate conclusions about functional brain activity. In addition, it typically has a time sampling rate of up to 10 Hz, which allows better tracking of the shape of the hemodynamic response function (Pinti et al., 2020).

VFTs are one of the commonly used executive function tasks, involving neuropsychological abilities such as verbal recall, retrieval, working memory, and attention (Whiteside et al., 2016). This task activates the prefrontal cortex, including the bilateral dorsolateral prefrontal cortex (DLPFC) (Seghier et al., 2004). Many studies have combined fNIRS and VFT to assess mental disorders, and VFT has been validated in the diagnosis of depression (Husain et al., 2020; Lee, Pavuluri, et al., 2019; Noda et al., 2017). fNIRS is very applicable and practical in predicting depression and monitoring disease progression (Li et al., 2022).

We hypothesize that low activation in the prefrontal lobe is a trait of patients with OSA, and the Oxy‐Hb response magnitude can distinguish the presence of depressive symptoms in OSA patients. Therefore, fNIRS was used in our study to investigate the cerebral cortical hemodynamics differences between OSA patients without depressive symptoms, OSA patients with depressive symptoms and HCs through VFT.

## 3 MATERIALS AND METHODS

### 3.1 Participants

This study included subjects attending sleep center of Beijing Tiantan Hospital from March 2021 to September 2022. The inclusion criteria of participants were as follows: ①18–80 years old; ② having ability to give informed consent. The exclusion criteria are as follows: ① having serious medical or surgical complications; ② having serious mental disorder, such as major depressive, bipolar, obsessive–compulsive, or posttraumatic stress; ③ currently receiving treatment with medications that may affect EEG recording, such as opioids, benzodiazepines, antidepressants, anticonvulsants, and antipsychotics;④ other sleep apnea diseases (central apnea, mixed apnea, periodic limb syndrome, insomnia, and others). The subjects arrived at the sleep laboratory before 18:30. We explained the research procedure to them before, and obtained their written consent after arrival. Baseline information, medical history, and scale assessment of these subjects were collected. All participants then completed fNIRS and polysomnography (PSG) that night. According to PSG results and 17‐item Hamilton Rating Scale for Depression (HAMD‐17) scores, subjects were divided into OSA patients with depressive symptoms (AHI ≥ 5 events/h, HAMD‐17 score >7), OSA patients without depressive symptoms (AHI ≥ 5 events/h, HAMD‐17 score < 7), and healthy subjects (AHI < 5 events/h, HAMD‐17 score ≤ 7). A total of 95 participants were included in our study: 31 OSA patients with depressive symptoms, 34 OSA patients without depressive symptoms, and 30 healthy controls (HCs). The three groups were matched for gender, age, and years of education. This study was approved by the Ethics Committee of Beijing Tiantan Hospital and conducted in accordance with the Declaration of Helsinki. All patients have signed written informed consent prior to participation in this study.

### 3.2 Functional near–infrared spectroscopy

**FIGURE 1:**
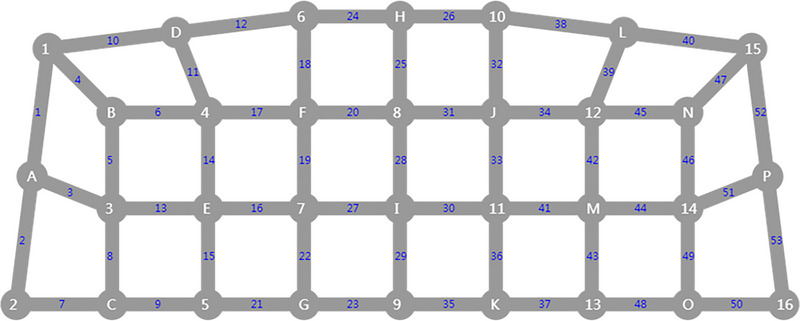
Optical fiber layout diagram. The arrangement of the photoelectric devices is based on the 10/20 system electrode placement method, which is also commonly used in electroencephalography. Optical fiber channels are distributed in the prefrontal lobe, with Channel 9 as the FPz reference point; the left and right cerebral hemispheres are symmetrically distributed along the T3–T4 line. There are 16 pairs of optoelectronic devices and 53 channels. The white number represents the sending probe, white letter represents the probing probe, and the blue number represents the channel.

**FIGURE 2:**
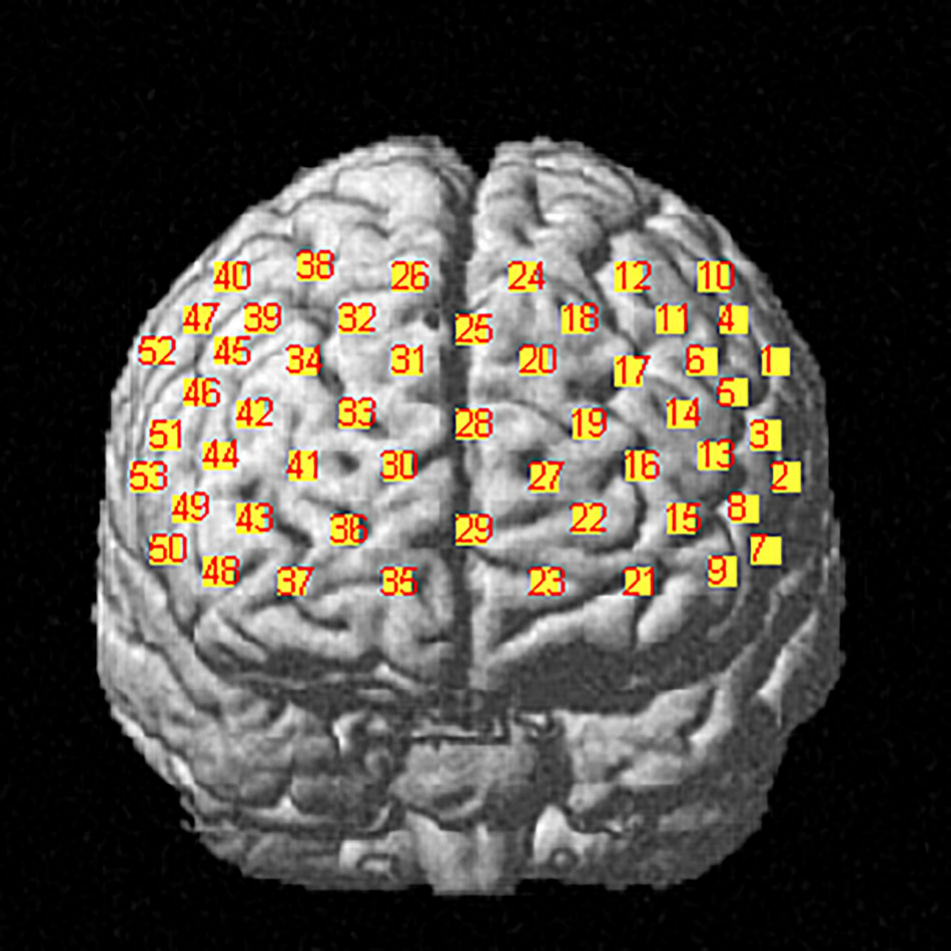
Channel location map. It shows that brain regions correspond to different channels of the functional near‐infrared spectroscopy (fNIRS). The number represents the channel.

Subjects were asked to sit comfortably in a quiet room for fNIRS examination prior to PSG. It used 53‐channel near‐infrared optical imaging system (BS‐7000, Wuhan Znion Technology Co., Ltd.) to measure the three relative concentration changes of Oxy‐Hb, Deoxy‐Hb, and Total‐Hb when completing VFT. The instrument has 16 pairs of transmitting and detecting probes. The wavelength of the emitted light is 690 and 830 nm, and the frequency is 100 Hz. The distance between each transmitter and the detector is 3 cm, and the area between them consists of a channel. Each probe is placed on the forehead. The arrangement of the photoelectric devices is shown in Figures 1 and 2, based on the 10/20 system electrode placement method, which is also commonly used in EEG (Okamoto et al., 2004).

### 3.3 Verbal fluency task

**FIGURE 3:**
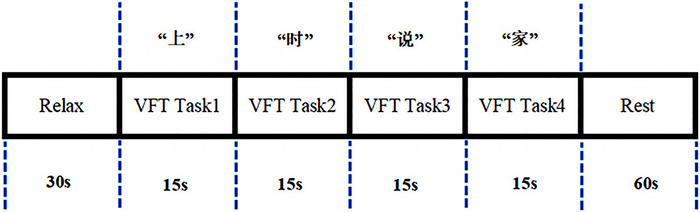
Experiment procedure. Verbal fluency task consists of a 30‐s pre‐task baseline, a 60‐s task period, and a 60‐s post‐task baseline. During the task, participants were asked to speak vocabulary as much as many and quickly as possible using four commonly used Chinese characters, such as “上,” “时,” “说,” and “家.” The four given characters changed every 15 s to stack signals for near‐infrared tasks.

As shown in Figure 3 , the VFT consists of a 30‐s pre‐task baseline, a 60‐s task period, and a 60‐s post‐task baseline. During the baseline period, participants were asked to repeat numbers according to instructions. During the task period, participants were asked not to move their heads but to keep their eyes fixed on a certain point and speak vocabulary as much as many and quickly as possible using four commonly used Chinese characters, such as “上,” “时,” “说,” and “家.” The four given characters changed every 15 s to stack signals for near‐infrared tasks.

### 3.4 Regions of interest (ROIs)

During VFT, there are usually changes in Oxy‐Hb of the prefrontal region, which is particular concerned (Ren et al., 2017; Yeung & Lin, 2021). The region of interest is set based on the channel distribution: premotor cortex and auxiliary motor cortex (detected by Channels 1, 4, 10, 40, 47, and 52), Broca's area (detected by Channels 2, 3, 5, 7, 8, 13, 44, 46, 49, 50, 51, and 53), DLPFC (detected by Channels 6, 11, 14, 17, 18, 20, 25, 31, 32, 34, 39, 42, and 45), frontal eye field (detected by Channels 12, 24, 26, and 38), and frontal polar region (detected by Channels 9, 15, 16, 19, 21, 22, 23, 27, 28, 29, 30, 33, 35, 36, 37, 41, 43, and 48).

### 3.5 PSG

Standard PSG recordings include six EEG leads (F3, F4, C3, C4, O1, and O2), one ECG channel, two ophthalmic channels, four myoelectric channels (bilateral mandibular and bilateral lower extremities), airflow through the nose and mouth recorded by thermal sensors, changes in body position recorded by position sensors, blood oxygen saturation recorded by fingertip oximeter, chest and abdominal breathing movements recorded by respiratory inductance plethysmography, and video surveillance. All PSG data were confirmed and reported by Registered Polysomnographic Technologists (RPSGTs) according to American Academy of Sleep Medicine (AASM) 2007 standards. Obstructive apnea is defined as airflow reducing by 80%–100% for at least 10 s and sustained breathing efforts in the chest and abdominal movement channels. AHI is calculated as the number of apnea and hypopnea events per hour among total sleep time. Patients whose apnea and hypopnea events are mainly obstructive with AHI ≥ 5/h are considered to have OSA (Peppard et al., 2013).

### 3.6 Assessment of depressive symptoms

HAMD‐17 is commonly used by clinicians to assess the severity of depressive symptoms. Seventeen items in HAMD‐17 were scored on a 5‐ or 3‐point scale, with a score of >7 being defined as the presence of depressive symptoms (Hamilton, 1960; Ren et al., 2016). OSA patients with HAMD‐17 score >7 were included in the group of OSA with depressive symptoms.

### 3.7 Data processing and statistical analysis

**FIGURE 4:**
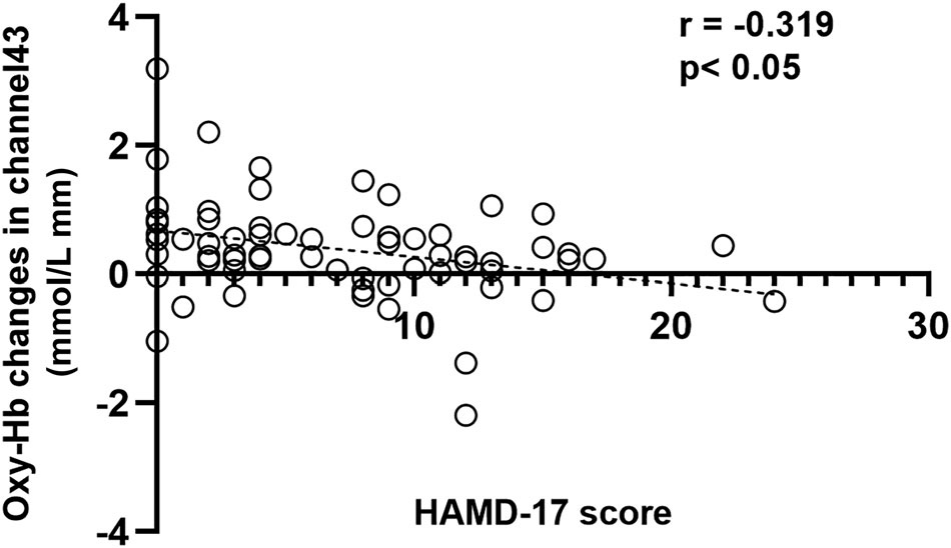
Associations between oxy‐hemoglobin (Oxy‐Hb) changes and 17‐item Hamilton Rating Scale for Depression (HAMD‐17) score in the obstructive sleep apnea (OSA) patients. A scatter plot graph shows the bivariate correlation of “Oxy‐Hb changes in Channel 43 vs. HAMD‐17 score” in the OSA patients. *p*‐Values indicate correlations between Oxy‐Hb changes and HAMD‐17 score. *r*, Spearman's *r*.

The normality of the data was checked by the Shapiro–Wilk test. One‐way analysis of variance was performed on the demographic data of the OSA without depressive symptoms group, the OSA with depressive symptoms group, and the HC group. Chi‐square test was used to compare the gender frequency among all groups. Because the continuous variables are not normally distributed, a non‐parametric test (Kruskal–Wallis *H* test) was used to assess the differences of PSG and fNIRS parameters among the three groups. Spearman's test was used to examine the correlation between HAMD‐17 score and various variables. GraphPad Prism 8 was used to generate Figure 4.

In the regression analysis, the HAMD‐17 score of OSA patients was used as the dependent variable of linear regression, and the following variables were input into the model: age, sex, BMI, PSG, and fNIRS–related parameters, using a stepwise regression model (excluding variables with *p* > .1). In order to solve the possible collinearity problem among the independent variables included in the regression analysis, the variance inflation factor (VIF) was tested, and the value of VIF > 5 was considered collinearity positive. The linear regression results are expressed as the β‐coefficient, its standard deviation, and the normalized coefficient, allowing quantification of the relative contribution of each covariate to the prediction of the HAMD‐17 score. *p* < .05 was considered to have statistical significance.

To determine the effect of cortical activation on depressive symptoms in OSA patients, we established a three‐level regression model using predictors to predict HAMD‐17 scores. The model consists of three variable input steps: In the first step, input demographic variables (age, gender); in the second step, enter PSG parameters related to HAMD‐17 score; finally, in Step 3, input near‐infrared‐related parameters into the model. Our aim was to explore whether near‐infrared related parameters were a larger part of the results than they were in the previous steps. This is reflected in the change in *R* ^2^ values (Δ*R* ^2^). Visual examination of the residual plot shows that the assumption of a linear relationship between the predictor and the outcome variable is adequately satisfied. All variables satisfy acceptable skewness and kurtosis ranges. The VIFs are between 1.014 and 1.078, indicating that the hypothesis of non‐multicollinearity is fully satisfied. Statistical analysis was conducted using SPSS 22.0.

Regarding fNIRS data, we mainly focus on the changes of Oxy‐Hb, because Oxy‐Hb has a superior signal‐to‐noise ratio and can reflect task‐related cortical activation more directly than Deoxy‐Hb. Therefore, we calculated the mean change in Oxy‐Hb activation across 53 channels by determining the difference during and before the mission. We used the Homer2 and NIRS‐KIT software packages to analyze fNIRS data. Data were preprocessed via the following steps. Motion artifacts were corrected by a moving source detector structure and a cubic spline interpolation method. A bandpass filter with cut‐off frequencies of 0–0.1 Hz was used to remove physiological noise. Oxy‐Hb was derived from optical densities using the modified Beer–Lambert law. VFT block waveforms were calculated with a block range set of 0–100 s, a pre‐baseline range set of 0–20 s, and a post‐baseline range set of 80–100 s. We used a 60 s task period of constructing phrases as the time window to analyze mean Oxy‐Hb changes. Linear fitting was applied to the data between these two baselines. The statistical results were corrected for multiple comparisons across channels by false discovery rate correction. All fNIRS data analysis procedures were accomplished under MATLAB R2013b.

## 4 RESULTS

### 4.1 Demographic characteristics and VFT performance

The demographic characteristics of subjects in each group are shown in Table 1. There were 30 males and 4 females in the OSA without depressive symptoms group (mean age 45.5 years, SD = 11.65), 25 males and 6 females in OSA with depressive symptoms (mean age 50.9 years, SD = 15.38), and 27 males and 3 females in the HCs (mean age 50.87 years, SD = 12.34). There was no significant difference in age (*H* = 3.46, *p* = .177), sex (*χ* ^2^ = 1.295, *p* = .523), and years of education (*H* = 4.47, *p* = .107) among these three groups (*p* > .05). The numbers of words produced in the VFT were also not statistically significant (*F* = 1.078, *p* = .345).

**TABLE 1:** Demographic characteristics, polysomnographic data, verbal fluency task (VFT) performance, and 17‐item Hamilton Rating Scale for Depression (HAMD‐17) scores among three groups.

	Health controls (*n* = 30)	OSA without depressive symptoms (*n* = 34)	OSA with depressive symptoms (*n* = 31)	*p*
**Demographic characteristics**				
Age	50.87 ± 12.34	45.5 ± 11.65	50.9 ± 15.38	.177^c^
Gender, female/male	3/27	4/30	6/25	.523^b^
Education, years	14.77 ± 3.42	14.44 ± 2.63	13.06 ± 4.02	.107^c^
BMI, kg/m^2^	24.64 ± 3.42	27.87 ± 4.6	26.23 ± 3.24	.005^a^
**Polysomnographic data**				
Sleep efficiency, %	75.39 ± 22.74	75.65 ± 17.11	73.09 ± 12.47	.121^c^
Sleep stage, %TST				
REM	16.63 ± 7.49	12.78 ± 6.81	16.23 ± 8.48	.085^a^
N1	18.38 ± 15.57	37.61 ± 23.98	23.37 ± 14.68	.001^c^
N2	57.44 ± 13.91	46.89 ± 21.16	54.94 ± 13.95	.035^a^
N3	7.55 ± 9.1	2.7 ± 5.02	5.47 ± 7.33	.009^c^
AHI, events/h	1.84 ± 1.39	37.19 ± 25.74	27.47 ± 22.8	*p* < .001^c^
Mean SpO_2_, %	96.5 ± 1.76	93.41 ± 5.3	95.01 ± 2.16	*p* < .001^c^
Lowest SpO_2_, %	92.2 ± 2.57	77.56 ± 13.89	85 ± 7.2	*p* < .001^c^
**VFT performance**	9.5 ± 4.34	8.24 ± 4.24	8.06 ± 3.96	.345^a^
**HAMD‐17 scores**	2.87 ± 2.24	2.26 ± 2.05	12.19 ± 3.95	*p* < .001^c^

*Note*: Values are expressed as mean ± SD [range]. *p*‐Values were derived from *χ* ^2^ test, one‐way ANOVA, or independent‐samples *t*‐test.

Abbreviation: OSA, obstructive sleep apnea.

^a^ Represents one‐way ANOVA.

^b^ Represents the *χ* ^2^ test.

^c^ Represents Kruskal–Wallis test.

### 4.2 Evaluation of polysomnography parameters and HAMD‐17 scale

Only AHI, mean SpO_2_, and lowest SpO_2_ were significantly different among the three groups (*p* < .001). Compared with HCs, OSA without depressive symptoms and OSA with depressive symptoms had higher AHI, lower mean SpO_2_, and lowest SpO_2_ (*p* < .001), but there were no significant differences in these parameters between OSA without depressive symptoms and OSA with depressive symptoms (*p* > .05).

HAMD‐17 scores (*H* = 63.302, *p* < .001) showed significant statistical differences among three groups. HAMD‐17 scores were lower in the OSA without depressive symptoms and HCs than the OSA with depressive symptoms group (*p* < .001). There was no significant difference between OSA with depressive symptoms and HCs (*p* > .05).

### 4.3 Average changes of Oxy‐Hb in VFT of the three groups and its correlation with HAMD‐17 score

The Oxy‐Hb changes of the three groups were significantly different in Channels 25 (*H* = 9.878, *p* = .007) and 43 (*H* = 6.957, *p* = .031). Further, inter‐group comparisons showed that the Oxy‐Hb change of Channel 25 (located in the dorsalateral prefrontal lobe) in OSA without depressive symptoms group was less than that in HCs group (*p* = .006), and the Oxy‐Hb change of Channel 43 (located in the right frontal pole) in OSA with depressive symptoms group was less than that in OSA without depressive symptoms group (*p* = .025). The average changes of Oxy‐Hb in each channel of the three groups are listed in Table 2.

**TABLE 2:** Channel‐to‐channel comparisons of mean oxy‐hemoglobin (Oxy‐Hb) (Mean ± SD).

				Mean Oxy‐Hb changes (mmol/L mm)			
Channel	Brodmann Area	Location	% Overlap	Health controls	OSA without depressive symptoms	OSA with depressive symptoms	*p* (FDR‐corrected)
1	6	Pre‐motor and supplementary motor cortex	0.743	0.51 ± 0.58	0.48 ± 0.99	0.27 ± 0.95	.193
2	44	Pars opercularis, part of Broca's area	0.54	0.87 ± 1.72	1.28 ± 1.02	0.13 ± 0.18	.343
3	45	Pars triangularis Broca's area	0.586	0.36 ± 1.08	0.5 ± 0.58	0.41 ± 1.03	.246
4	6	Pre‐motor and supplementary motor cortex	0.413	0.21 ± 1.32	0.28 ± 1.09	0.1 ± 1.52	.655
5	45	Pars triangularis Broca's area	0.653	0.55 ± 1.7	0.37 ± 0.92	−0.01 ± 1.2	.217
6	9	Dorsolateral prefrontal cortex	0.343	0.17 ± 0.82	−0.01 ± 0.64	0.03 ± 0.63	.171
7	45	Pars triangularis Broca's area	0.97	0.77 ± 1.02	0.6 ± 0.71	0.7 ± 1.4	.735
8	45	Pars triangularis Broca's area	0.902	0.48 ± 1.33	0.76 ± 0.91	0.55 ± 0.97	.413
9	46	Dorsolateral prefrontal cortex	0.917	0.29 ± 0.6	0.32 ± 0.45	0.05 ± 0.34	.072
10	6	Pre‐motor and supplementary motor cortex	0.537	0.25 ± 1.39	0.04 ± 1.06	0.23 ± 1.09	.493
11	9	Dorsolateral prefrontal cortex	0.924	0.37 ± 1.75	0.18 ± 0.67	−0.16 ± 0.81	.393
12	8	Includes frontal eye fields	0.705	0.2 ± 0.6	0.03 ± 0.97	−0.15 ± 0.67	.131
13	45	Pars triangularis Broca's area	0.621	−0.12 ± 1.02	0.27 ± 0.7	0.03 ± 2.36	0.057
14	46	Dorsolateral prefrontal cortex	0.736	−0.13 ± 0.91	0.18 ± 0.65	0.39 ± 3.03	.668
15	46	Dorsolateral prefrontal cortex	0.54	0.43 ± 0.53	0.41 ± 0.44	1.32 ± 3.99	.864
16	10	Frontopolar area	0.515	0.19 ± 0.39	0.18 ± 0.91	1.22 ± 4.39	.988
17	9	Dorsolateral prefrontal cortex	0.527	0.33 ± 0.82	0.3 ± 0.73	0.13 ± 0.75	.413
18	9	Dorsolateral prefrontal cortex	1	0.43 ± 1.6	0.3 ± 1.4	0.19 ± 0.8	.783
19	10	Frontopolar area	0.526	0.08 ± 0.5	−0.002 ± 0.36	0.02 ± 0.38	.445
20	9	Dorsolateral prefrontal cortex	0.88	0.2 ± 0.64	0.08 ± 0.41	0.34 ± 0.45	.104
21	10	Frontopolar area	0.523	0.37 ± 0.51	0.19 ± 0.56	0.85 ± 2.76	.783
22	10	Frontopolar area	0.909	0.4 ± 0.75	0.35 ± 0.85	0.52 ± 2.01	.921
23	10	Frontopolar area	0.571	0.44 ± 0.53	0.4 ± 0.57	1.02 ± 3.27	.255
24	8	Includes frontal eye fields	0.605	0.31 ± 0.61	0.17 ± 0.83	0.04 ± 0.63	.255
25	9	Dorsolateral prefrontal cortex	0.966	0.27 ± 0.44	0.01 ± 0.34	0.21 ± 0.37	**.007**
26	8	Includes frontal eye fields	0.629	0.07 ± 0.54	0.08 ± 0.3	0.08 ± 0.4	.653
27	10	Frontopolar area	1	0.38 ± 0.75	0.27 ± 0.59	0.2 ± 0.56	.798
28	10	Frontopolar area	0.935	0.19 ± 0.44	0.13 ± 0.77	0.15 ± 0.55	.302
29	10	Frontopolar area	1	0.19 ± 0.43	0.11 ± 0.32	0.16 ± 0.39	.32
30	10	Frontopolar area	1	0.2 ± 0.34	0.23 ± 0.53	0.15 ± 0.43	.68
31	9	Dorsolateral prefrontal cortex	0.866	0.22 ± 0.61	0.21 ± 0.5	0.2 ± 0.68	.82
32	9	Dorsolateral prefrontal cortex	0.983	0.1 ± 0.88	0.33 ± 0.94	0.36 ± 1.1	.621
33	10	Frontopolar area	0.59	0.08 ± 0.42	0.07 ± 0.51	0.09 ± 0.29	.792
34	9	Dorsolateral prefrontal cortex	0.69	0.18 ± 0.67	0.19 ± 0.58	0.13 ± 0.41	.779
35	10	Frontopolar area	0.615	0.35 ± 0.46	0.28 ± 0.62	0.1 ± 0.39	.084
36	10	Frontopolar area	0.885	0.23 ± 0.45	0.23 ± 0.58	0.28 ± 0.33	.85
37	11	Orbitofrontal area	0.545	0.04 ± 1.8	0.44 ± 1.1	0.19 ± 0.78	.46
38	8	Includes frontal eye fields	0.796	0.09 ± 0.73	0.02 ± 0.65	0.33 ± 0.52	.084
39	9	Dorsolateral prefrontal cortex	0.933	0.21 ± 0.61	−0.01 ± 0.84	0.04 ± 0.44	.181
40	9	Dorsolateral prefrontal cortex	0.588	0.27 ± 1.23	0.3 ± 1.82	0.19 ± 0.61	.409
41	10	Frontopolar area	0.655	0.25 ± 0.42	0.27 ± 0.54	0.3 ± 0.81	.714
42	46	Dorsolateral prefrontal cortex	0.772	0.29 ± 0.59	0.3 ± 0.53	0.13 ± 0.51	.255
43	10	Frontopolar area	0.539	0.42 ± 0.48	0.61 ± 0.77	0.14 ± 0.71	**.031**
44	45	Pars triangularis Broca's area	0.584	0.3 ± 0.41	0.33 ± 1.1	0.2 ± 0.56	.851
45	9	Dorsolateral prefrontal cortex	0.507	0.33 ± 0.46	0.01 ± 0.75	0.03 ± 0.41	.13
46	45	Pars triangularis Broca's area	0.689	0.22 ± 0.48	0.41 ± 1.15	0.23 ± 0.58	.821
47	9	Dorsolateral prefrontal cortex	0.478	1.67 ± 6.4	0.45 ± 3.99	−1.29 ± 7.95	.263
48	46	Dorsolateral prefrontal cortex	0.868	0.25 ± 0.63	0.2 ± 0.44	0.2 ± 0.74	.794
49	45	Pars triangularis Broca's area	0.832	−0.07 ± 3.87	0.8 ± 1.57	0.58 ± 1.08	.707
50	45	Pars triangularis Broca's area	0.971	0.03 ± 3.34	1 ± 0.92	1.03 ± 2.55	.153
51	45	Pars triangularis Broca's area	0.687	0.49 ± 0.78	0.45 ± 1.22	0.37 ± 0.84	.636
52	6	Pre‐motor and supplementary motor cortex	0.793	0.34 ± 0.91	0.31 ± 1.01	0.29 ± 1.09	.454
53	44	Pars opercularis, part of Broca's area	0.49	0.83 ± 0.95	0.94 ± 1.1	0.7 ± 1.37	.493

Abbreviations: FDR, false discovery rate; OSA, obstructive sleep apnea.

### 4.4 Linear regression analyses of factors associated with HAMD‐17 score in OSA patients

For the OSA patients, we found significant negative correlations between HAMD‐17 scores and mean Oxy‐Hb changes in Channel 43 (*r* = −.319, *p* < .05) (see Figure 4). Linear regression after adjusting for confounding factors, such as age, gender, and BMI (Table 3), showed that only non‐rapid eye movement (NREM) Stage 1 sleep (N1)% (*t* = −3.089, *p* = .003) was retained in the final model. The regression model had statistical significance (*F* = 9.54, *p* < .01), but the adjusted *R* ^2^ of the model was .132. So the regression model could explain 13.2% of the variation of the dependent variable HAMD‐17 score, and the explanatory power of the model was slightly insufficient.

**TABLE 3:** Univariate and multivariate linear regression analyses of factors associated with 17‐item Hamilton Rating Scale for Depression (HAMD‐17) score in obstructive sleep apnea (OSA) patients.

	Univariate analysis			Multivariate analysis		
Characteristics	Beta Coefficient	*p*	95% CI	Standardized coefficient	*p*	VIF
**Demographic and clinical characteristics**						
Age	.043	.736	−.219 to .301			
Gender	.042	.738	−.219 to .317			
Education	−.202	.106	−.443 to .052			
BMI	−.21	.093	−.477 to .079			
**Polysomnographic data**						
Sleep efficiency, %	−.242	.052	−.468 to .061			
Sleep stage, %TST						
REM	.166	.185	−.081 to .429			
N1	−.32	**.009**	−.555 to −.072	−.363	**.003**	
N2	.171	.174	−.063 to .408			
N3	.189	.132	−.008 to .133			
AHI, events/h	−.267	**.031**	−.478 to −.029			
Mean SpO_2_, %	.212	.09	−.042 to .433			
Lowest SpO_2_, %	.289	**.02**	.013–.52			
**fNIRS data**						
VFT performance	−.085	.503	−.353 to .168			
Oxy‐Hb changes in Channel 13	−.215	.085	−.432 to .048			
Oxy‐Hb changes in Channel 43	−.319	**.01**	−.526 to −.074			

*Note*: Bold font indicates variables with statistically significant differences between clusters (*p* < .05).

Abbreviations: AHI, apnoea‐hypopnoea index; BMI, body mass index; fNIRS, functional near‐infrared spectroscopy; HAMD‐17, 17‐item Hamilton Rating Scale for Depression; Lowest SpO_2_, Lowest oxygen saturation; Mean SpO_2_, Mean oxygen saturation; N1, non‐REM (NREM) Stage 1; N2, non‐REM (NREM) Stage 2; N3, non‐REM (NREM) Stage 3; Oxy‐Hb, oxy‐hemoglobin; REM, rapid eye movement; TST, total sleep time; VFT, verbal fluency task; VIF, variance inflation factor.

### 4.5 Hierarchical regression predicting HAMD‐17 scores in OSA patients

The hierarchical regression model summaries for HAMD‐17 scores can be found in Table 4. Step 1 of the hierarchical regression analysis entered demographic variables into the model. This step did not account for a significant proportion of the variance in HAMD‐17 scores (Adj. *R* ^2 ^= −.018; *F*[2,92] = .17, *p* = .844). Step 2 entered the block of PSG features (N1%, AHI, and Lowest SpO_2_) into the model. The model at Step 2 contained demographic variables, and three PSG feature variables. This step accounted for an increase in the proportion of the variance in HAMD‐17 scores accounted for by the model at Step 1 (Δ*R* ^2 ^= .103). Step 3 entered Oxy‐Hb changes in Channel 43 into the model. The model at Step 3 included demographic variables, three PSG feature variables, and Oxy‐Hb changes in Channel 43. This step accounted for an increase in the proportion of the variance in HAMD‐17 scores accounted for by the model at Step 2 (Δ*R* ^2 ^= .05). These results suggest that Oxy‐Hb changes in Channel 43 account for a significant proportion of the variation, above and beyond that accounted for by demographic variables and PSG features. A summary of this analysis can be found in Table 4.

**TABLE 4:** Regression steps and analysis of variance (ANOVA) summaries of hierarchical model predicting 17‐item Hamilton Rating Scale for Depression (HAMD‐17) scores in obstructive sleep apnea (OSA) patients.

Step and predictor variable		*B*	*β*	CI	Adj. *R* ^2^	Δ*R* ^2^
**Step 1**:						
Age		.008	.02	−.075, .091	−.018	.004
Gender		.899	.058	−2.309, 4.107		
**Step 2**:						
N1		−.095	−.36	−.167, −.023	.057	.103
AHI		.094	.436	.022, .166		
Lowest SpO_2_		10.694	.221	−4.377, 25.766		
**Step 3**:						
Oxy‐Hb changes in Channel 43		−.177	−.229	−.333, −.223	.099	.05
**ANOVA model summary**						
**Model**		**SS**	**df**	**Mean square**	***F***	**Sig**
1	Regression	9.947	2	4.974	.17	.844
	Residual	2690.2	92	29.241		
	Total	2700.147	94			
2	Regression	288.747	5	57.749	2.131	.069
	Residual	2411.4	89	27.094		
	Total	2700.147	94			
3	Regression	422.423	6	70.404	2.72	.018^**^
	Residual	2277.724	88	25.883		
	Total	2700.147	94			

Abbreviations: Adj.R^2^, the explanatory power of the model; ANOVA, analysis of variance; CI, confidence interval; df, degrees of freedom; Oxy‐Hb, oxy‐hemoglobin; ΔR^2^, model fit degree changes; SS, sum of squares.

** *p* < .05.

The final model at Step 3, containing all predictor variables, explained a significant proportion of the variance in HAMD‐17 scores (Adj. *R* ^2 ^= .099, *F*[1,88] = 5.165, *p* = .018). Results indicate that for every one SD decrease in Oxy‐Hb changes in Channel 43 (mmol/L mm), there is a corresponding .229 SD increase in depressive symptoms when all other variables are held constant, although the effect is small.

## 5 DISCUSSION

Our study is the first to use fNIRS to compare differences in prefrontal activation between OSA patients and healthy subjects, suggesting that altered hemodynamic responses may be a potential mechanism for executive dysfunction in OSA patients. At the same time, this study provides evidence for fNIRS as a potential tool to predict depressive symptoms in OSA patients. Hierarchical regression models indicated that Oxy‐Hb changes in Channel 43 were a significant negative associate of the depressive symptoms in OSA patients. Importantly, this association was seen while controlling for other PSG features associated with the HAMD‐17 scores.

Many previous studies have used magnetic resonance imaging to explore brain function in OSA patients. Studies have shown that DLPFC participates in the ventral attention network and is active in response to unexpected stimuli (Corbetta et al., 2000, 2002, 2008). A study of functional connectivity in the brain network at rest state found that the right DLPFC of OSA patients showed a reduction in resting‐state functional connectivity, suggesting a functional deficit (Zhang et al., 2013). In a study that simultaneously recorded muscle sympathetic nerve activity and fMRI of the brain, it was found that left and right DLPFC signal changes were enhanced in OSA patients with muscle vasoconstriction‐driven elevation (Fatouleh et al., 2014). A multimodal meta‐analysis of structural and fMRI studies found that patients with OSA exhibited low activation in the DLPFC compared to healthy population controls (Huang et al., 2019). The above results are basically consistent with the conclusion of our study.

Moreover, we found, for the first time, that OSA patients with depressive symptoms had less cortical activation in the channel of right frontal polar region than those without depressive symptoms. Previous fNIRS studies have also found that depressed adolescents show less cortical activation than healthy subjects in channels located primarily in the prefrontal cortex (Liu et al., 2022). A study examining the effects of music therapy on brain activity in patients with major depression found that patients had significantly lower average Oxy‐Hb values than HCs (Feng et al., 2019). A systematic review of depression detection using near‐infrared spectroscopy and verbal fluency tests has found low activation in frontal regions of major depression disorder (Yeung & Lin, 2021). Assessment of depression in OSA patients can be confusing because of overlapping symptoms between these two disorders (Bucks et al., 2018). Therefore, the results of our study are helpful to clarify the mechanism of depression symptoms in OSA patients. Our results further suggest that lower levels of cortical activation in frontal polar region may be an important associate of depressive symptoms in an OSA population. Thus, reduced cortical activity in frontal polar region may constitute a specific biomarker of OSA patients’ depressive symptoms.

The results of our study showed that AHI and lowest SpO_2_ were negatively associated with depressive symptoms, which is consistent with the results of some recent studies (Bjorvatn et al., 2018; Lee, Yoon, et al., 2019; Luik et al., 2015). However, some previous studies showed a positive correlation between sleep apnea severity and depressive symptoms (Dai et al., 2016; Edwards et al., 2015). In addition, some studies failed to find any association between depressive symptoms and the severity of OSA (Garbarino et al., 2020). Compared with the self‐rating scale used in these studies, the scale we used evaluated by professionals is more objective.

The linear regression model in our study ultimately retained only N1% to predict the HAMD‐17 score. Previous studies on abnormal sleep in depression mainly focused on sleep parameters in the REM period. A study on abnormal sleep structure in adolescents found that REM sleep latency was an important predictor of the onset of major depression (Ma et al., 2023). However, another study found that patients with depression also had an increase in N1 compared to HCs (Asaad et al., 2016). And there was also a study that showed that people with bipolar disorder who have suicidal ideation have more NREM Stage 1 sleep (Bernert et al., 2017). Therefore, it is possible that parameters of NREM Stage 1 sleep may also act as predictors of depression.

The current study has several limitations. First, due to the cross‐sectional design of our study, we were unable to explore potential predictors of longitudinal prognosis. Therefore, near‐infrared spectroscopy measurements should be performed during long‐term follow‐up to investigate predictive biomarkers associated with clinical symptoms and functional outcomes. Second, the subjects included in our study were assessed by the scale only for depressive symptoms. Future studies can include people with depression through the diagnosis of DSM‐5, and fNIRS can be used to identify the differences in internal brain activity between OSA patients with depression and OSA patients without depression, which is helpful for clinical diagnosis, treatment, and prognosis. Third, the gender ratio in each group is quite uneven, which might affect the generalization of the findings. Additionally, all the sleep parameters used in this study are conventional, and quantitative analysis of sleep EEG can be introduced for further research.

## 6 CONCLUSION

The altered prefrontal hemodynamic response may be a potential mechanism for executive dysfunction in OSA patients. In addition, fNIRS may be a potential tool for predicting depressive symptoms in OSA patients.

## AUTHOR CONTRIBUTIONS

**Xuan Zhang**: Writing—original draft; writing—review and editing. **Ning Zhang**: Conceptualization. **Yang Yang**: Methodology; data curation; formal analysis; supervision. **Shuo Wang**: Validation; investigation; software. **Ping Yu**: Visualization; project administration. **Chun‐Xue Wang**: Resources; writing—review and editing; supervision.

## CONFLICT OF INTEREST STATEMENT

The authors declare no conflicts of interest.

### PEER REVIEW

The peer review history for this article is available at https://publons.com/publon/10.1002/brb3.70038.

## Data Availability

The data that support the findings of this study are available from the corresponding author upon reasonable request.
